# Exponentially decaying modes and long-term prediction of sea ice concentration using Koopman mode decomposition

**DOI:** 10.1038/s41598-020-73211-z

**Published:** 2020-10-01

**Authors:** James Hogg, Maria Fonoberova, Igor Mezić

**Affiliations:** 1 Aimdyn, Inc., Santa Barbara, CA, 93101 USA; 2 University of California, Santa Barbara, USA

**Keywords:** Cryospheric science, Applied mathematics, Nonlinear phenomena

## Abstract

Sea ice cover in the Arctic and Antarctic is an important indicator of changes in the climate, with important environmental, economic and security consequences. The complexity of the spatio-temporal dynamics of sea ice makes it difficult to assess the temporal nature of the changes—e.g. linear or exponential—and their precise geographical loci. In this study, Koopman Mode Decomposition (KMD) is applied to satellite data of sea ice concentration for the Northern and Southern hemispheres to gain insight into the temporal and spatial dynamics of the sea ice behavior and to predict future sea ice behavior. We observe spatial modes corresponding to the mean and annual variation of Arctic and Antarctic sea ice concentration and observe decreases in the mean sea ice concentration from early to later periods, as well as corresponding shifts in the locations that undergo significant annual variation in sea ice concentration. We discover exponentially decaying spatial modes in both hemispheres and discuss their precise spatial extent, and also perform predictions of future sea ice concentration. The Koopman operator-based, data-driven decomposition technique gives insight into spatial and temporal dynamics of sea ice concentration not apparent in traditional approaches.

## Introduction

Sea ice is floating ice that forms when ocean water freezes. The formation and distribution of sea ice plays an important role in the planet’s climate and thus large amounts of data related to quantitative measures of sea ice have been collected, including continuous satellite remote sensing measurements since 1978. The decreasing extent of Arctic sea ice over the last several decades has had negative effects on Arctic wildlife and local communities, while also potentially opening new regions to maritime commerce and natural resources exploration. The future of sea ice behavior is thus of great significance for environmental, economic, and national security reasons. There are several studies that suggest a nonlinear trend in the decline of the sea ice cover^1,2^.

A variety of approaches have been applied to predict future sea ice behavior over short time scales (1–3 months in the future), including both dynamical (model-based, either coupled ice-ocean-atmosphere or ice-ocean models) and statistical (data-based) (see the reports of the Sea Ice Prediction Network (SIPN)^3^ and, e.g. kernel analog forecasting^4^). Statistical approaches were reported by the SIPN to be similarly or more successful compared with dynamical approaches for prediction of sea ice distributions 1–3 months in the future^3^. Of statistical approaches, spatial mode based approaches^5^ have particular advantages. Compared to regression or trend based approaches, spatial mode based approaches are powerful tools for studying and predicting the geographic and temporal behavior of sea ice because they decompose the time dependent sea ice data into time varying spatial structures of physical significance. However, such approaches have heretofore been restricted to methods that—explicitly or implicitly—assume statistical stationarity of the underlying process. Here we apply Koopman Mode Decomposition (KMD)^6,7^—a method capable of capturing nonstationary, growing or decaying trends in data—to sea ice concentration dynamics and prediction.

KMD is a mathematical tool well suited to analyzing sea ice dynamical behavior because it identifies important spatial structures and their complex time dependence from large data sets such as those available for sea ice. The Koopman operator theory^7–13^ is already widely used to analyze data and provide models for complex dynamic processes. Mathematically, the Koopman operator^14^ is a linear representation on an observables space of an appropriate group action on state space. As it is a linear representation, it is natural that key objects of analysis will be its eigenvalues and eigenfunctions. It turns out that in distributed systems (for example, those with a spatial component, e.g. a fluid velocity field, or a dynamical system on a graph) in addition to the eigenvalues and eigenfunctions, there is a third class of objects of importance: the Koopman modes^7, 13^ which are projections of fields of observables on eigenfunctions of the Koopman operator^7^. The eigenvalues of the Koopman operator provide the time scales on which—a potentially exponential—change in sea ice cover is happening, and the modes indicate the spatial extent of the changes. Crucially, the Koopman operator methods do not require a model—observables like sea ice thickness and concentration are sufficient to compute the eigenvalues and the associated modes.

The most popular computational method for Koopman eigenvalues and modes is the Dynamic Mode Decomposition (DMD), that has become a major tool in the data-driven analysis of complex dynamical systems. DMD was first introduced in 2008 by P. Schmid^15^ for the study of fluid flows where it was conceptualized as an algorithm to decompose the flow field into component fluid structures, called “dynamic modes” or “DMD modes”, that described the evolution of the flow. The DMD modes and their temporal behavior are given by the spectral analysis of the linear operator, which is constructed from data since it is assumed that direct access to it is not available. The book^16^ provides references and introduction to a variety of DMD-related algorithms. Rowley et al.^13^ gave the method theoretical underpinnings by connecting it to the spectral analysis of the Koopman operator. The paper^17^ provides both enhancements and analysis to the DMD method as well as additional theoretical underpinning for its relationship to the Koopman operator.

**Figure 1:**
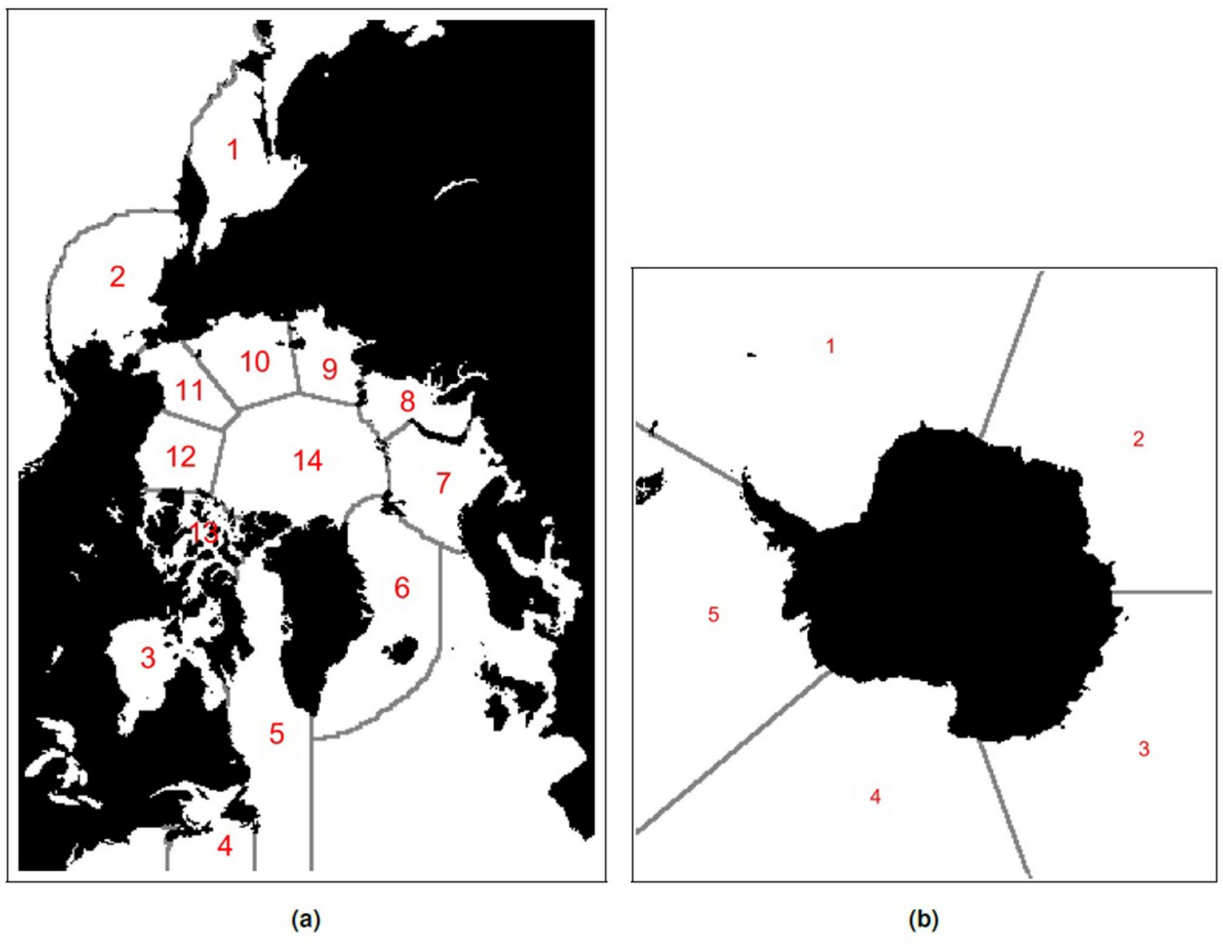
Northern and Southern hemisphere geographic regions. (**a**) Northern hemisphere geographic regions. 1: Sea of Okhotsk, 2: Bering Sea, 3: Hudson Bay, 4: Gulf of St Lawrence, 5: Baffin Bay, 6: East Greenland Sea, 7: Barents Sea, 8: Kara Sea, 9: Laptev Sea, 10: East Siberian Sea, 11: Chukchi Sea, 12: Beaufort Sea, 13: Canadian Arctic Archipelago, 14: Central Arctic Ocean. (**b**) Southern hemisphere geographic regions. 1: Weddell Sea, 2: Indian Ocean, 3: Pacific Ocean, 4: Ross Sea, 5: Bellingshausen and Amundsen Seas.

A data set well suited for study and prediction with KMD is the satellite-based sea ice concentration measurements from the NSIDC Sea Ice Index^18^, due primarily to the long and continuous time period (from November 1978 to the present) and the large geographic regions over which this data is available. KMD analysis was applied to both entire Arctic and Antarctic sea regions. The geographic regions mentioned below for the Arctic were those given by Boisvert and Stroeve^19^ and the Antarctic regions were those given by the NSIDC^18^ (see Fig. 1 for the definitions of each region). Note that using these geographic definitions, not all of the ocean region in the Northern hemisphere data is considered in the Arctic, therefore the sea ice concentration data from these non-Arctic regions were excluded from the KMD analysis.

Examination of a mode shows the geographic locations where the sea ice concentration has oscillatory, growth or decay behavior as determined by the associated eigenvalue, and thus allows one to associate particular Koopman modes with aspects of sea ice dynamics of interest. For example, modes with oscillatory frequencies with periods of one year correspond to the annual variation between the sea ice minimum extent in the late summer and the sea ice maximum extent in the late winter, and modes with eigenvalues near zero correspond to the mean sea ice concentration over the time period spanned by the data. Furthermore, the presence of eigenvalues with multi-year oscillatory periods suggests the presence of long-term periodic variations in sea ice behavior, and eigenvalues with real components leading to relatively slow growth or decay time constants suggests the existence of long-term increases or decreases in sea ice concentration, where again the associated modes show the geographic regions where such behaviors occur.

The spectral expansion of the Koopman operator shows that in a large class of systems, the evolution can be described by a combination of complex exponential and algebraic terms^6^.

The Koopman modes and eigenvalues also permit prediction of the future sea ice concentration behavior. Data decomposed using KMD can be reconstructed over its original time period, and these same reconstruction equations allow for prediction of future behavior simply by increasing the time variable to values beyond the time period of the original input data. In this work we apply KMD to data over various multi-year time windows before a given year and produce prediction results for after the time window for which data was available, thus enabling a judgment of the goodness of the KMD predictions by comparison with the true “future” values and calculation of forecast skill by comparison with four reference models.

Examination of the skill of KMD-based prediction using short and long input data periods compared with various reference models gives information about the relative dominance of cyclo-stationary dynamics versus nonlinear variation in the multi-year hemispheric-level behavior of sea ice concentration. For systems with dynamics dominated by cyclo-stationary variation, the KMD prediction will be related to the climatological mean, as the dynamics evident in the input data consist of oscillatory behavior. In contrast, for systems dominated by non-stationary dynamics such as long-term nonlinear climate changes, KMD can be expected to detect the exponential growth/decay behavior present in input data^7^. In this case a KMD-based prediction is expected to outperform predictions based on long-term averages, such as a climatological forecast, and to have closer to equivalent skill compared with predictions based on means taken over shorter time periods.

The primary results of the work are:

- The presence of Koopman modes showing the change in geographic distribution of sea ice over the time period covered by satellite data, specifically the reduction in the mean Arctic and Antarctic sea ice concentration and the increased annual variation near West Antarctica and in the Arctic marginal seas.
- Long-term exponential decay behavior in sea ice concentration in both the Arctic and Antarctic that is indicative of feedback mechanisms that accelerate decline in the extent of the sea ice cover^1,20,21^.
- Qualitatively different skill values found for Koopman-based prediction of future Arctic and Antarctic sea ice behavior, where the skill for Arctic sea ice concentration compared with climatological models was marginally positive, but the skill for Antarctic predictions was roughly equal. We suggest this may show dominance, on a hemispheric level, of nonlinear dynamics in the Northern hemisphere and cyclo-stationary dynamics in the Southern hemisphere.

## Results

We have performed KMD processing on the sea ice concentration and anomaly data using both Arnoldi-type and DMD-based KMD algorithms^7,13,15,17^ to analyze the resulting Koopman modes and eigenvalues. All of the algorithms used gave very similar results. This suggests that the sea ice concentration data dynamical behavior is “well behaved” in the sense that the resulting condition number is sufficiently small that any of the various approximations of the Koopman decomposition are valid here^17^ and thus supports the conclusion that the KMD results obtained here are physically meaningful and not numerical artifacts.

### Koopman modes

**Figure 2:**
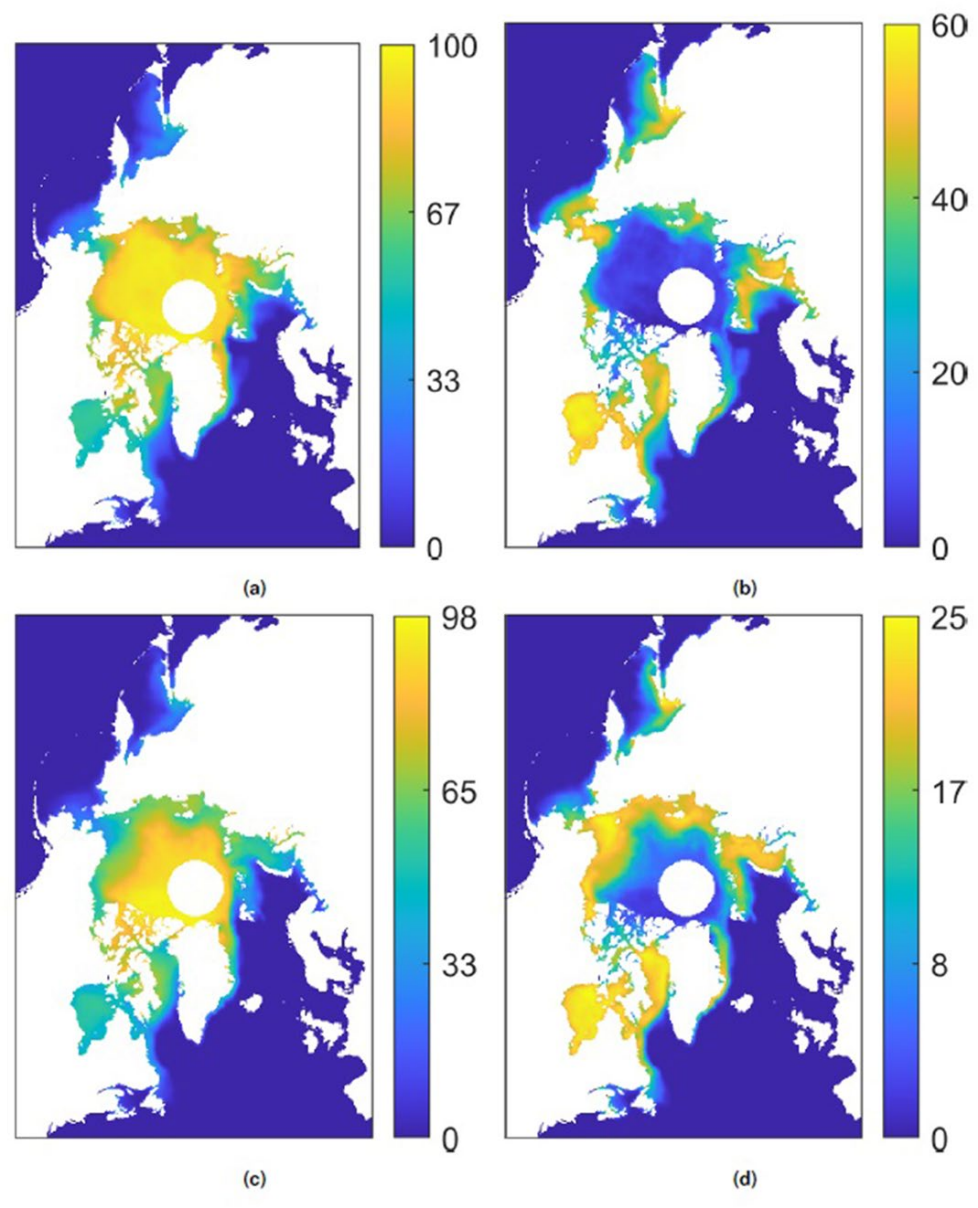
Koopman modes representing the mean and annual variation in sea ice concentration over five year windows for the Northern hemisphere. (**a**) Mean coverage, 1979–1983 period, (**b**) annual variation, 1979–1983 period, (**c**) mean coverage, 2014–2018 period, (**d**) annual variation, 2014–2018 period. The colorbar units are percent concentration.

**Figure 3:**
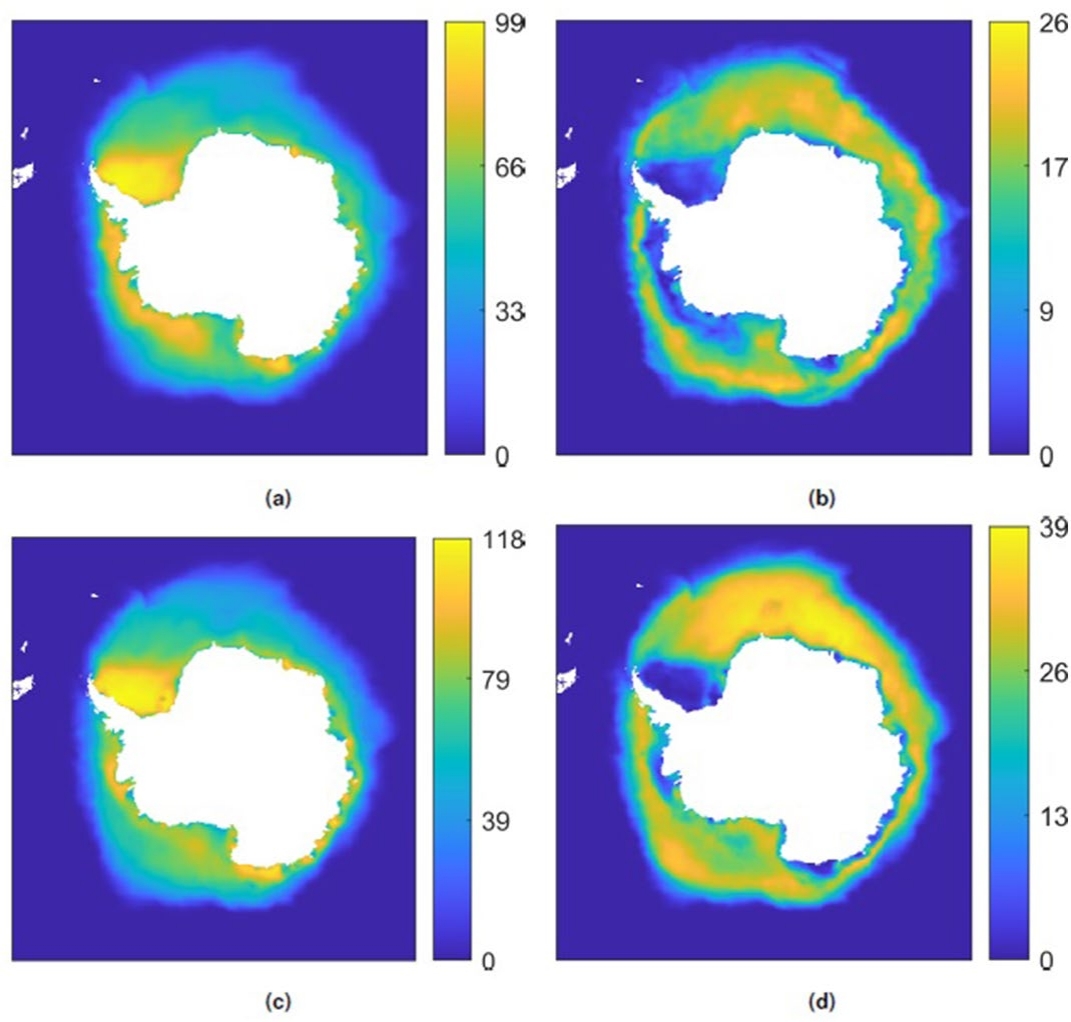
Koopman modes representing the mean and annual variation in sea ice concentration over five year windows for the Southern hemisphere. (**a**) Mean coverage, 1979–1983 period, (**b**) annual variation, 1979–1983 period, (**c**) mean coverage, 2014–2018 period, (**d**) annual variation, 2014–2018 period. The colorbar units are percent concentration; see the note in the text regarding mode elements taking on non-physical values.

Figures 2 and 3 show Koopman modes corresponding to the mean and annual variation in sea ice concentration for two 5-year time periods (1979–1983 and 2014–2018), as well as modes corresponding to long-term exponential decay over a 40-year time period. The mean mode in each case is defined as that with the largest L2-norm (taken over the components of the mode) and whose corresponding eigenvalue has zero or negligible real and imaginary components. The annual mode in each case is the mode with a $\tau ^{osc}$ value closest to 12 months. In all cases the annual mode was unambiguously identifiable as a large L2-norm mode. The long-term exponential decay modes shown for the 40-year window are the two largest L2-norm modes with $\tau ^{decay}$ periods greater than one year. Note that although the modes have the same units as the input data, the modes can include non-physical values (i.e. concentration values greater than 100%) because the Koopman Mode Decomposition is a non-orthogonal decomposition, so the modes, which are projections of observables onto eigenfunctions, are not necessarily equal to or less than the magnitudes of the observables. Consider the simple case of a vector (0, *a*) with $|a|<1$. This can be decomposed as $(0,a) = (-1,0) + (1,a)$, where both “modes” $(-1,0)$ and (1, *a*) have norm greater than *a*.

Comparison of the mean modes in Fig. 2a with Fig. 2c, and Fig. 3a with Fig. 3c, shows that the mean sea ice concentration is lower in the later time periods, suggesting that the sea ice does not reach as great an extent in the winter. Similarly, examination of the annual variation modes in the two time periods shows a significant shift in which geographic regions have the largest amplitude mode components, with many of the Arctic marginal seas now having large amplitude mode components indicating greater annual variation in sea ice concentration. This greater annual variation is particularly in the regions of the Beaufort Sea, Kara Sea, and the coastal corridor in between, and in the Bellingshausen and Amundsen Seas near West Antarctica and the Pacific Ocean in East Antarctica, with relatively little change in the Ross Sea area.

The combination of the mean and annual variation modes shown in Figs. 2 and 3 can be viewed as first-order models of the sea ice concentration dynamics in each hemisphere over short annual timescales during the respective five-year windows. The observed decreases in sea ice concentration from the earlier to later periods suggests that a mode with primarily long-time decaying behavior is needed to reproduce the long-term loss of sea ice observed in the regions identified above.

**Figure 4:**
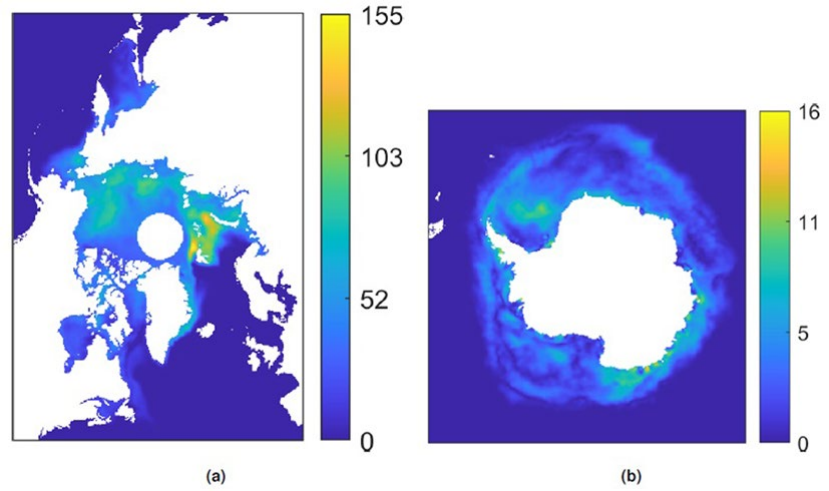
Koopman modes representing long term, exponential decay for the period 1979–2018. (**a**) Koopman mode representing long term, exponential decay in the Northern hemisphere, corresponding to exponential decay with $\tau _{decay} = 131 \,\hbox {months}$. (**b**) Koopman mode representing long term, exponential decay in the Southern hemisphere, corresponding to exponential decay in the Antarctic with $\tau _{decay} = 234 \,\hbox {months}$. The colorbar units are percent concentration; see the note in the text regarding mode elements taking on non-physical values.

Such modes are apparent in analysis of the entire 40 year period of the data set. Figure 4a shows such a mode from the Northern hemisphere for the entire 40 year data set with $\tau _{decay} = 131 \,\hbox {months}$ and no oscillatory component. Consistent with other observations^22–24^, these modes show that the decrease in sea ice coverage is most pronounced in the regions of the Beaufort Sea and Barents Sea. This is also consistent with the changes in the mean and annual variation observed in the five-year window cases above. Figure 4b shows an equivalent mode for the Southern hemisphere, with slow decay ($\tau _{decay} = 234 \,\hbox {months}$) and long oscillation period ($\tau ^{osc} = 238 \,\hbox {months}$) representing a decrease in sea ice concentration, primarily consisting of a decrease in sea ice concentration in West Antarctica. This region is known to be warming more rapidly than the region as a whole^25,26^, so this KMD mode is consistent with that observation and the result from the five-year windows above showing decreased mean ice coverage and increased annual variation near West Antarctica. Note that the exponential decay described by the identified modes occurs in the geographic regions indicated by the spatial content of the mode, so the decrease in sea ice concentration on those time scales occurs locally in those regions.

The identification of an oscillatory period comparable to or greater than half of the time period of the input observations (that is, 238 months as described above, compared to the 480 months of available input data) is believed to have dynamical significance despite this frequency being less than the frequency resolution that would be obtainable from the discrete Fourier transform (DFT) of this time series data. As described in greater detail in the Appendix included in the Supplementary information, KMD does not have a fixed frequency resolution, unlike the DFT, and the multiple spatial dimensions of the input data effectively permits sampling of the underlying system dynamics over a wider range of oscillation phase values than would observation of the time variation of a single spatial point. We hypothesize that the oscillatory dynamics observed in the system will include adequate phase differences between spatial positions to expose the observed low frequency information to the KMD algorithm.

### Forecast model predictions

As described in the Methods section below, forecasts of future sea ice concentration can be made by “recomposition” of the Koopman Mode Decomposition, where a subset of the eigenvalues and modes obtained from performing KMD over a given time period of data are used to calculate predicted concentration or anomaly values for times after the time period of the input data. As described in more detail in the Methods section, the goodness of the forecasts is described by a skill calculation, where the KMD-based forecast is compared to one of four different reference models: (1) a climatological model, in which each future calendar month is forecast to be the mean of the data values of that calendar month for the entire period of available sea ice concentration data, (2) a persistence model, in which the anomaly of each future month is forecast to be the same as in a reference month, (3) a climatological-type model in which the forecast for each future month is the mean of that calendar month in the input data (rather than the entire period of available data as in the climatological model), and (4) a linear fit of the data values for each calendar month in the input data.

In the following, the skill, root mean square error (RMSE) and anomaly correlation coefficient (ACC) results comparing the KMD-based prediction to the climatological and persistence models are shown first, as those are standard reference models used for skill calculation. Results are then shown for the remaining two reference models to contrast the resulting skill values and their implications for the nature of the dominant system dynamics.

**Figure 5:**
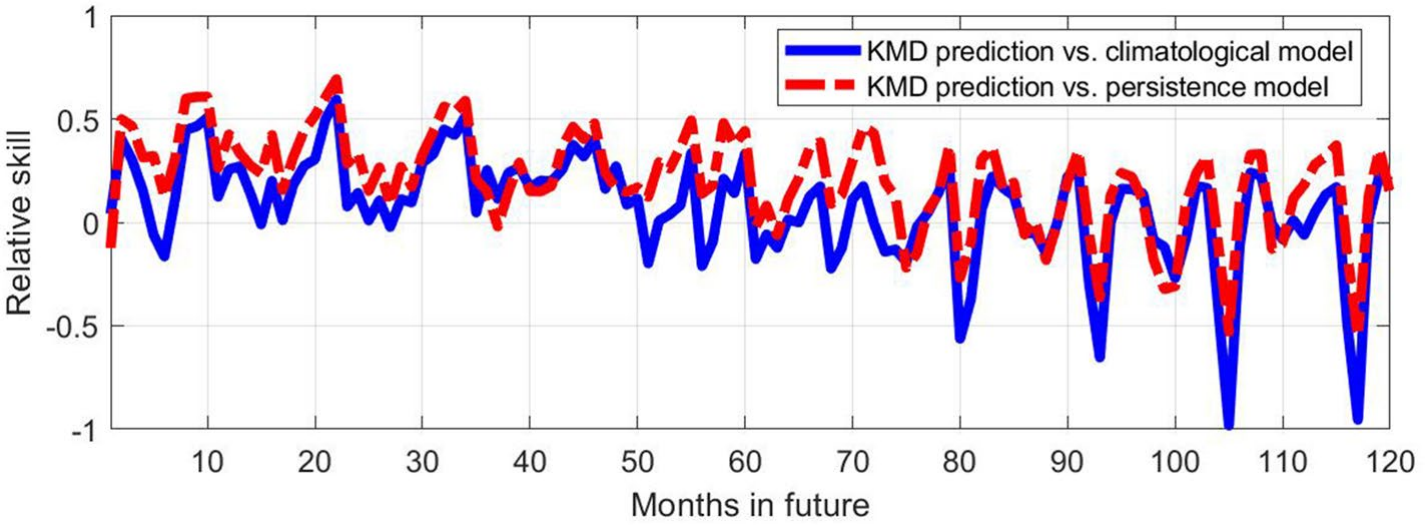
Relative skill values for ten future years for the KMD prediction vs. the climatological model (solid blue line) and vs. the persistence model (dot-dashed red line), for the Northern hemisphere and for KMD applied to the 30 year period 1979–2008.

**Figure 6:**
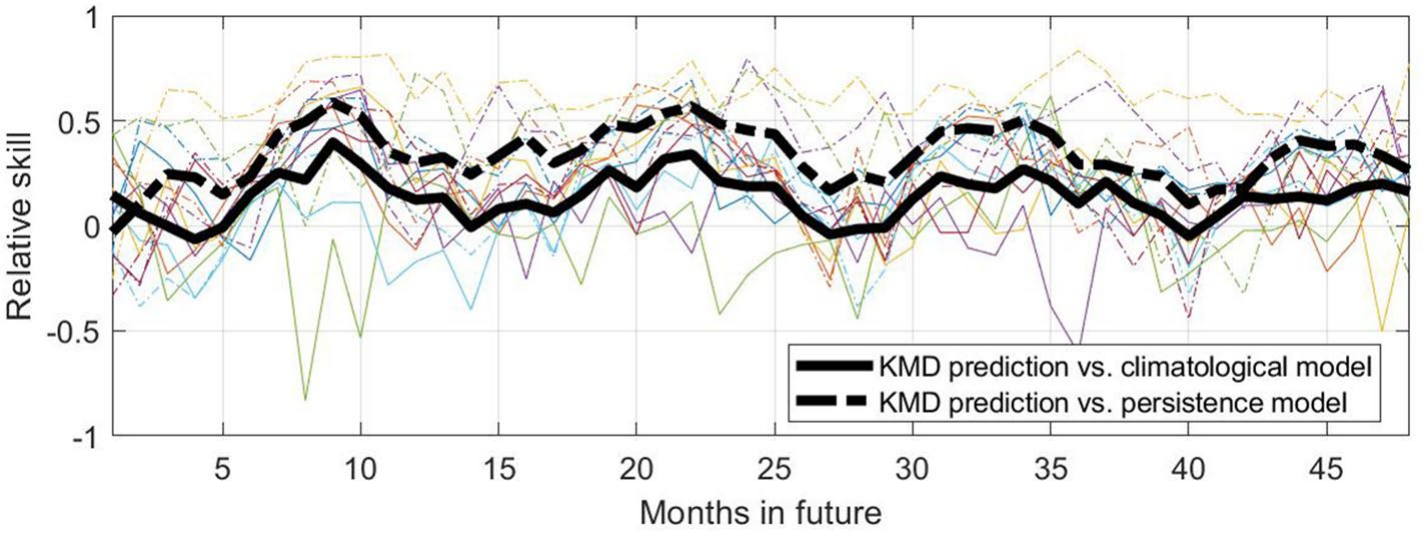
Relative skill values for four future years for the KMD prediction vs. the climatological model (solid lines) and vs. the persistence model (dot-dashed lines), for the Northern hemisphere and for KMD applied to seven 30-year periods (1979–2008, 1980–2009, etc.). The thin, colored lines are monthly skill values for each of the seven input periods and the thick black lines are the mean of the monthly skill values for all the seven input periods.

**Figure 7:**
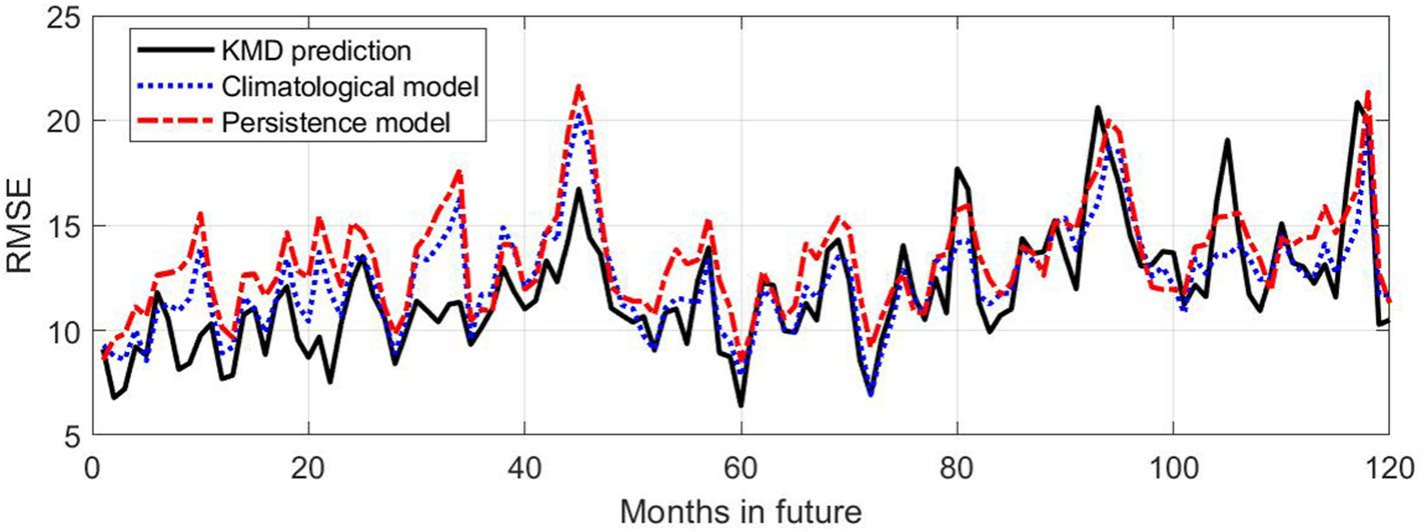
Root mean square error (RMSE) values (in percent concentration) for ten future years for the KMD prediction (solid black line), the climatological model (dotted blue line) and the persistence model (dot-dashed red line), for the Northern hemisphere and for KMD applied to the 30 year period 1979–2008.

**Figure 8:**
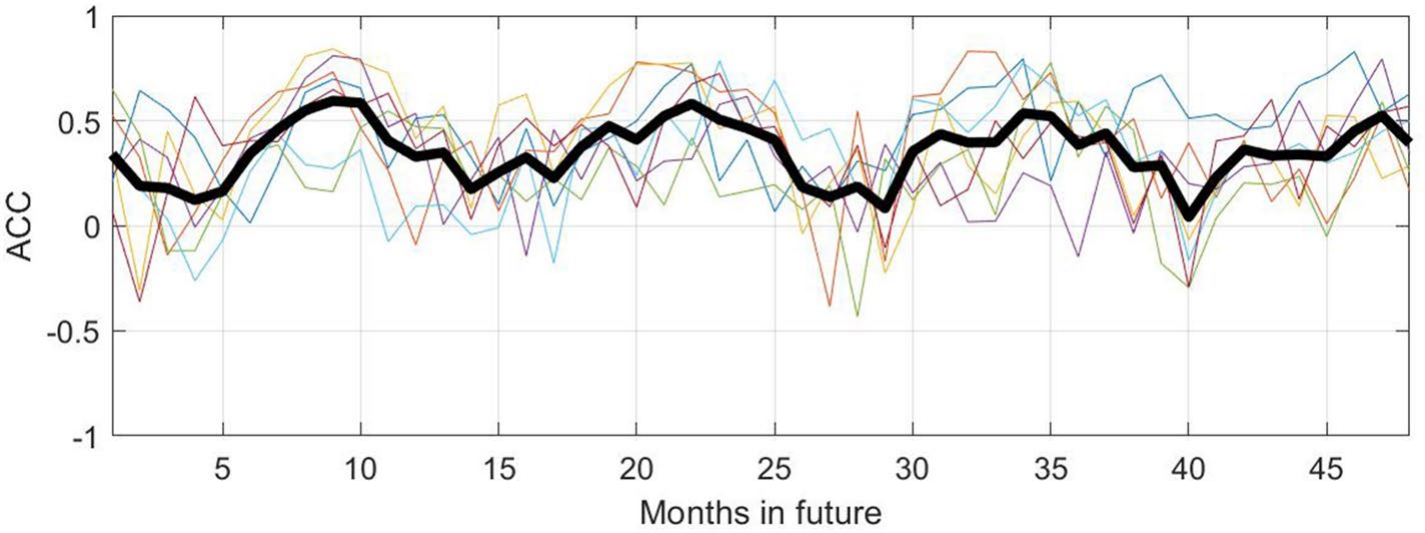
Anomaly correlation coefficient (ACC) values for four future years for the KMD prediction, for the Northern hemisphere and for KMD applied to seven 30-year periods (1979–2008, 1980–2009, etc.). The thin, colored lines are monthly ACC values for each of the seven input periods and the thick black line is the mean of the monthly ACC values for all of the seven input periods.

**Figure 9:**
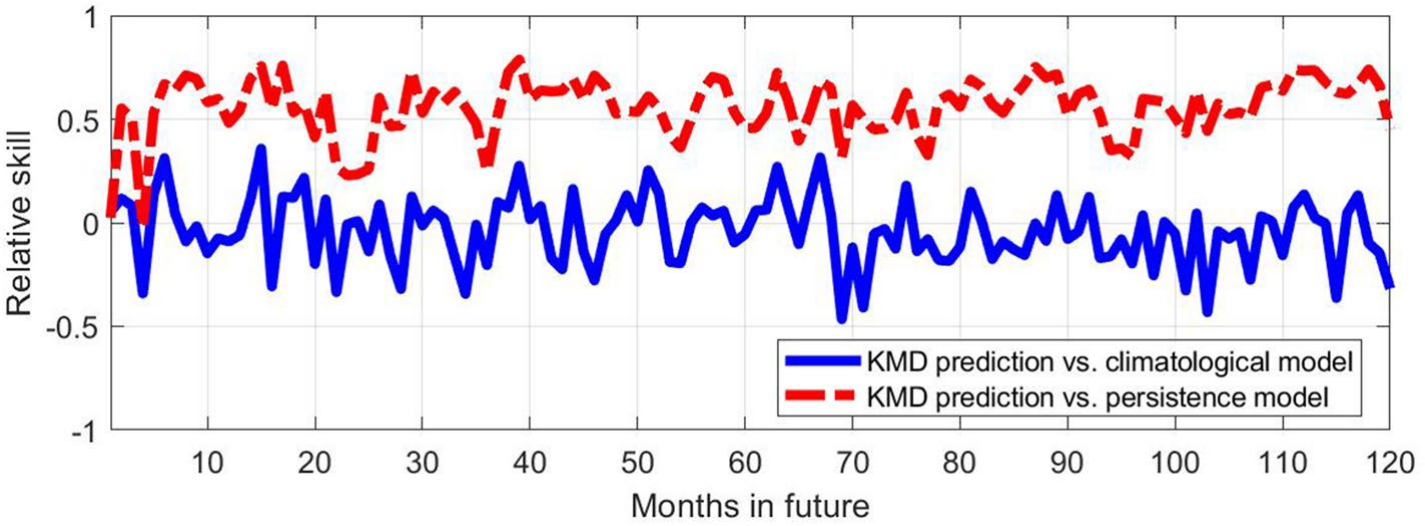
Relative skill values for ten future years for the KMD prediction vs. the climatological model (solid blue line) and vs. the persistence model (dot-dashed red line), for the Southern hemisphere and for KMD applied to the 30 year period 1979–2008.

**Figure 10:**
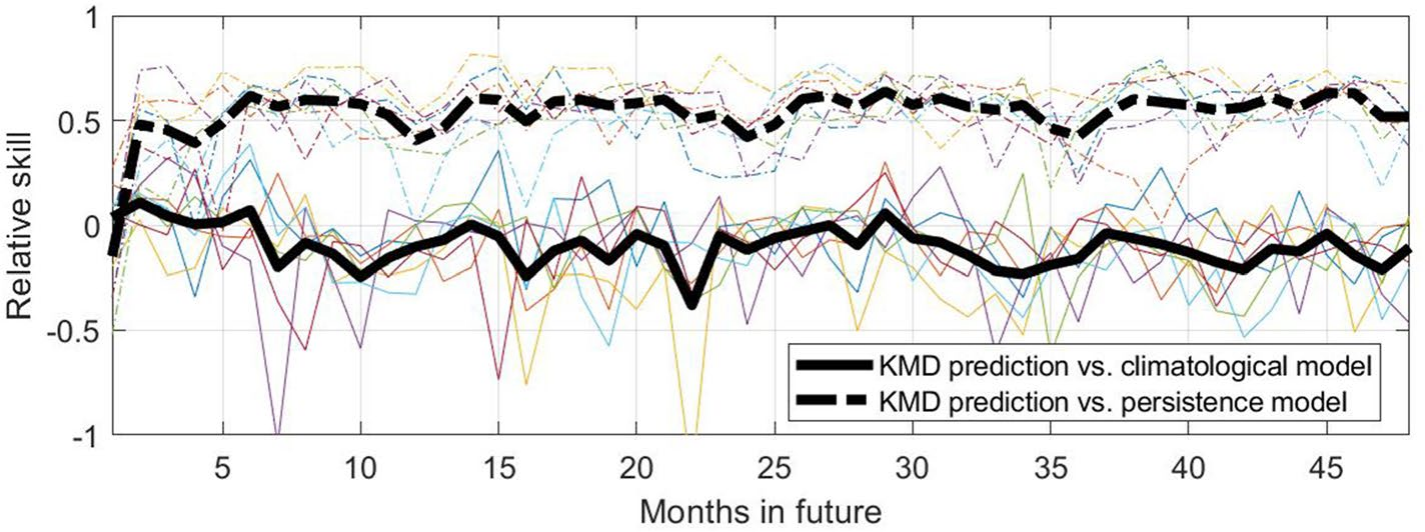
Relative skill values for four future years for the KMD prediction vs. the climatological model (solid lines) and vs. the persistence model (dot-dashed lines), for the Southern hemisphere and for KMD applied to seven 30-year periods (1979–2008, 1980–2009, etc.). The thin, colored lines are monthly skill values for each of the seven input periods and the thick black lines are the mean of the monthly skill values for all the seven input periods.

**Figure 11:**
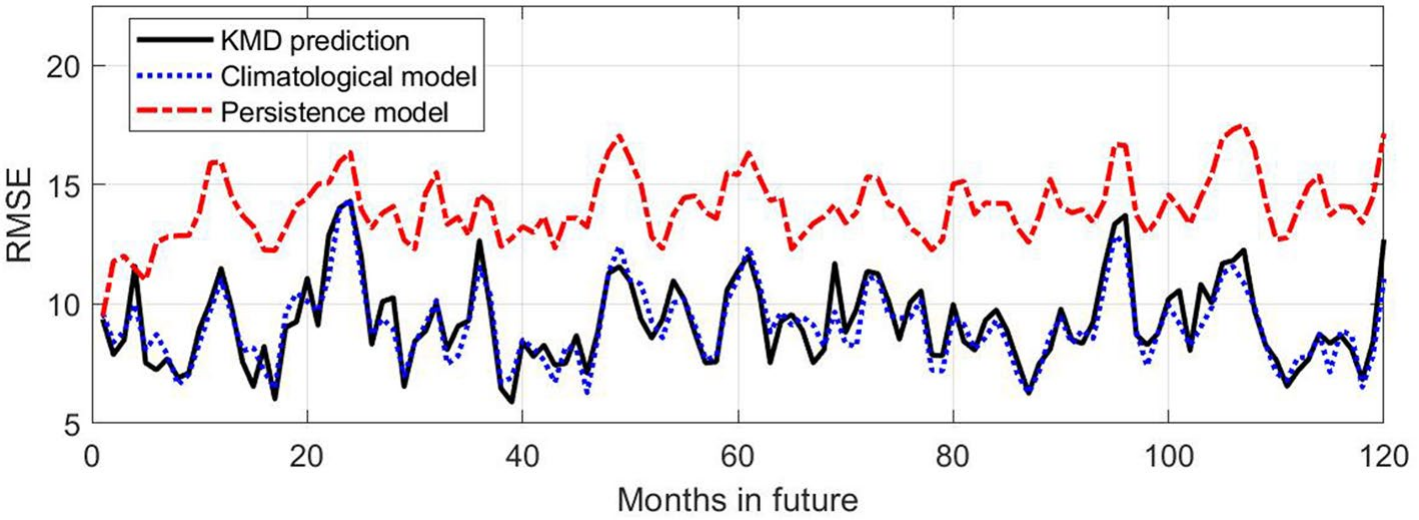
Root mean square error (RMSE) values (in percent concentration) for 10 future years for the KMD prediction (solid black line), the climatological model (dotted blue line) and the persistence model (dot-dashed red line), for the Southern hemisphere and for KMD applied to the 30 year period 1979–2008.

**Figure 12:**
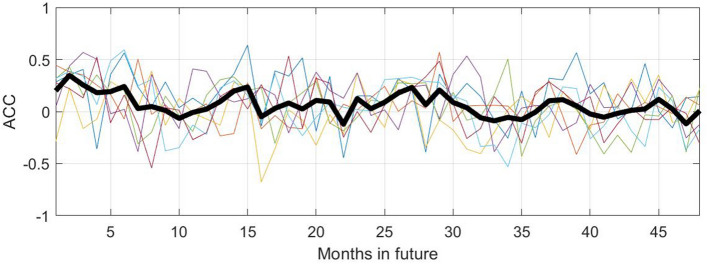
Anomaly correlation coefficient (ACC) values for four future years for the KMD prediction, for the Southern hemisphere and for KMD applied to seven 30-year periods (1979–2008, 1980–2009, etc.). The thin, colored lines are monthly ACC values for each of the seven input periods and the thick black line is the mean of the monthly ACC values for all of the seven input periods.

Figure 5 shows relative skill values (as defined in Eq. 5) for the KMD forecast compared with the climatological and persistence models, for KMD applied to sea ice anomaly values from January 1979 to December 2008 and with forecasts 10 years beyond that period, beginning in January 2009. As described in the Methods section, maximum skill values were obtained when using a subset of the Koopman modes and eigenvalues for forming the forecast. The results shown in Figs. 5, 6, 7, 8, 9, 10, 11, 12 use the 16 (out of 29) largest mode and eigenvalue pairs when sorted by their residual^17^. It is seen in Fig. 5 that the skill versus both the climatological and persistence models are almost uniformly positive until approximately 48 months into the future, and they vary on an apparently annual time scale with peak skill values around the summer sea ice minima.

Figure 6 shows similar skill values over a shorter forecast period of 48 months beyond the input data time period. Here KMD is applied to 30 year windows of sea ice anomaly values, for seven consecutive time windows (1979–2008, 1980–2009, etc.), and the skill for each case for the 48 months beyond in the respective input periods are shown together, where the thick black lines show the means of the seven traces for the skill based on each of the two reference models. The behavior noted previously in Fig. 5 for the results from a single input data period is seen to be relatively consistent for the seven different input data periods, where despite some scatter in the individual traces the trend of positive skill with peaks during the summer sea ice minima is apparent. We note that the forecast results are shown beginning one month in the future (i.e., $s=1$ as described in the Methods section), so while the skill versus the persistence model will be very poor at $s=0$, after one month the sea ice anomaly values have changed sufficiently to improve the skill value.

Figure 7 shows the root mean square error (RMSE) values derived from the mean square error (MSE) values used to calculate the skill values shown in Fig. 5. Consistent with the skill results, the KMD prediction error is consistently lower than the reference model errors until approximately 48 months into the prediction period, beyond which the KMD prediction has comparable error to the reference models. The persistence model has, as expected, comparatively low error at the beginning of the prediction period but almost immediately rises to have the largest error of the three models. The anomaly correlation coefficient (or score) is shown in Fig. 8 for the four-year prediction periods following the seven 30-year KMD input data windows described above. There is seen to be positive correlation for the mean ACC and most of the individual traces for the four year period, however they generally only exceed the synoptically useful threshold of 0.5 or 0.6 for the summer sea ice minima during this period.

Figures 9, 10, 11, 12 show the skill, RMSE, and ACC results for the Southern hemisphere. Strikingly different behavior compared to the Northern hemisphere case is evident in this case, where the KMD prediction is seen to perform more poorly after the first month of the prediction period. The errors of the KMD prediction and climatological model are consistently nearly identical and the mean ACC values remain below 0.5.

**Figure 13:**
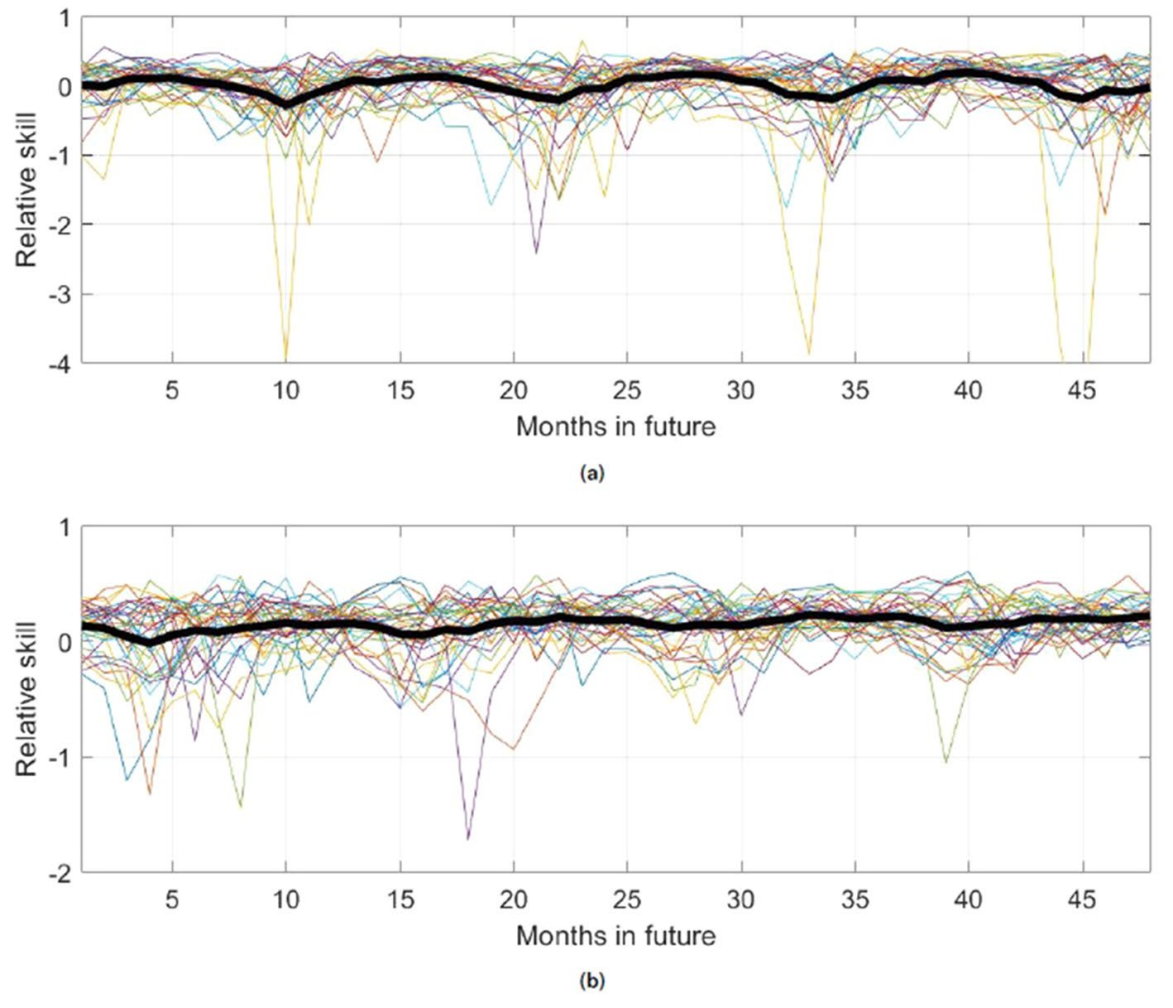
Relative skill for KMD prediction versus the mean of input data model for each month of the four year future prediction period, subsequent to each four year period of past input data. The thin, colored lines are monthly skill values for each four year input period and the thick black line is the mean skill of each input period for that number of months past the input time period. (**a**) Northern hemisphere, (**b**) Southern hemisphere.

**Figure 14:**
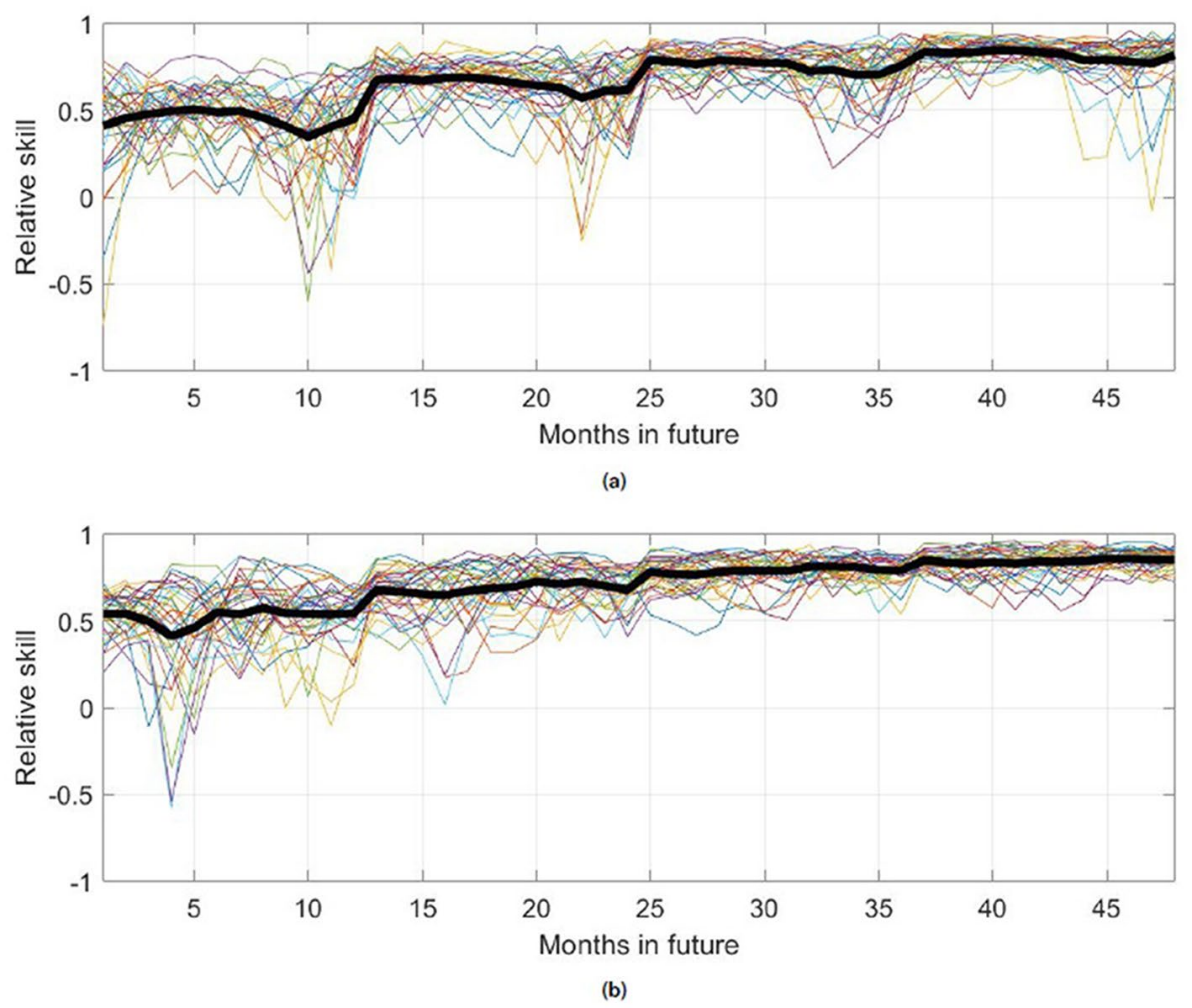
Relative skill for KMD prediction versus the linear fit model for each month of the four year future prediction period, subsequent to each four year period of past input data. The thin, colored lines are monthly skill values for each four year input period and the thick black line is the mean skill of each input period for that number of months past the input time period. (**a**) Northern hemisphere, (**b**) Southern hemisphere.

**Figure 15:**
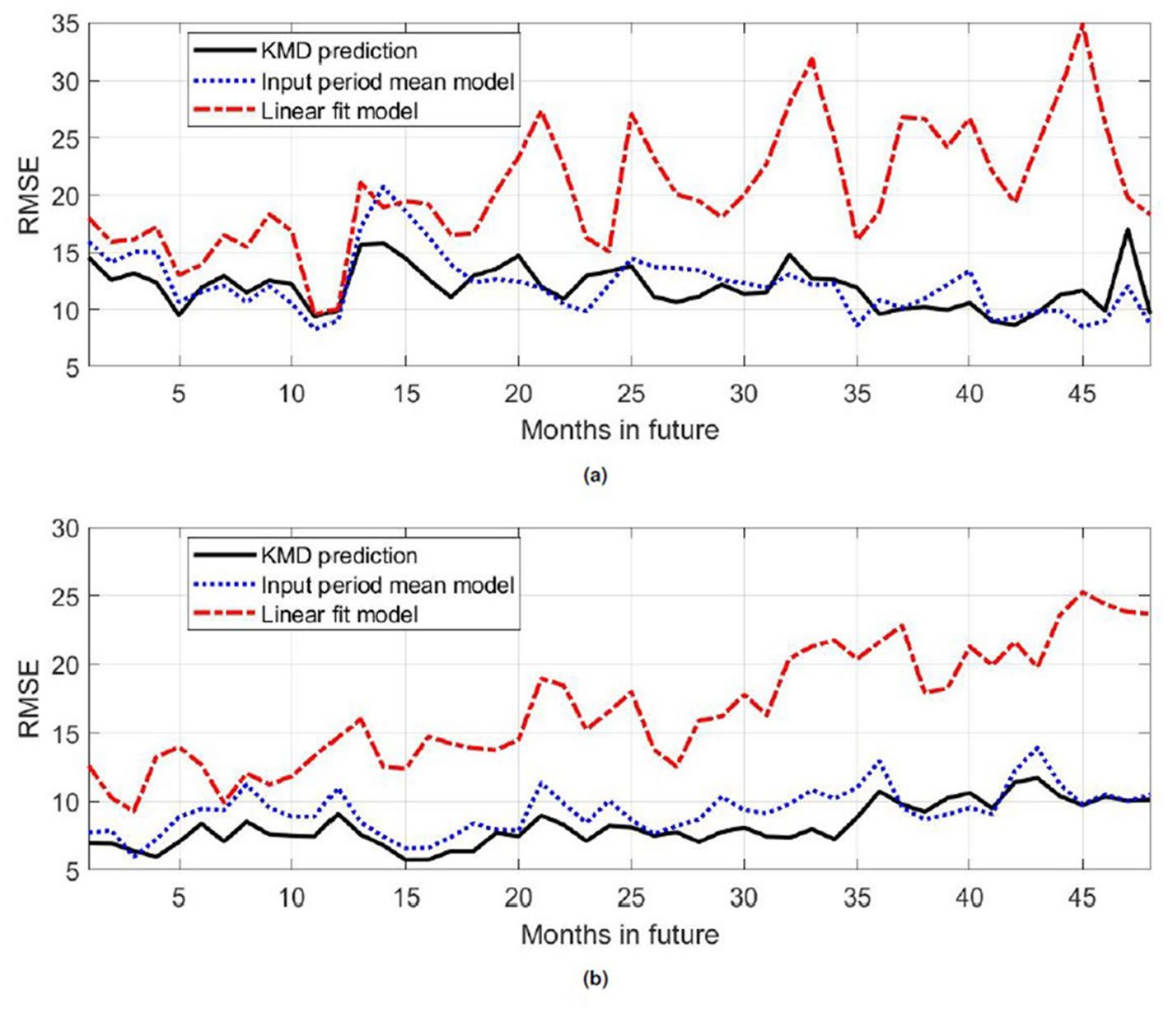
Root mean square error (RMSE) values (in percent concentration) for four future years for the KMD prediction (solid black line), the input period mean model (dotted blue line) and the linear fit model (dot-dashed red line), for KMD applied to the four year periods. (**a**) Northern hemisphere, (**b**) Southern hemisphere.

**Figure 16:**
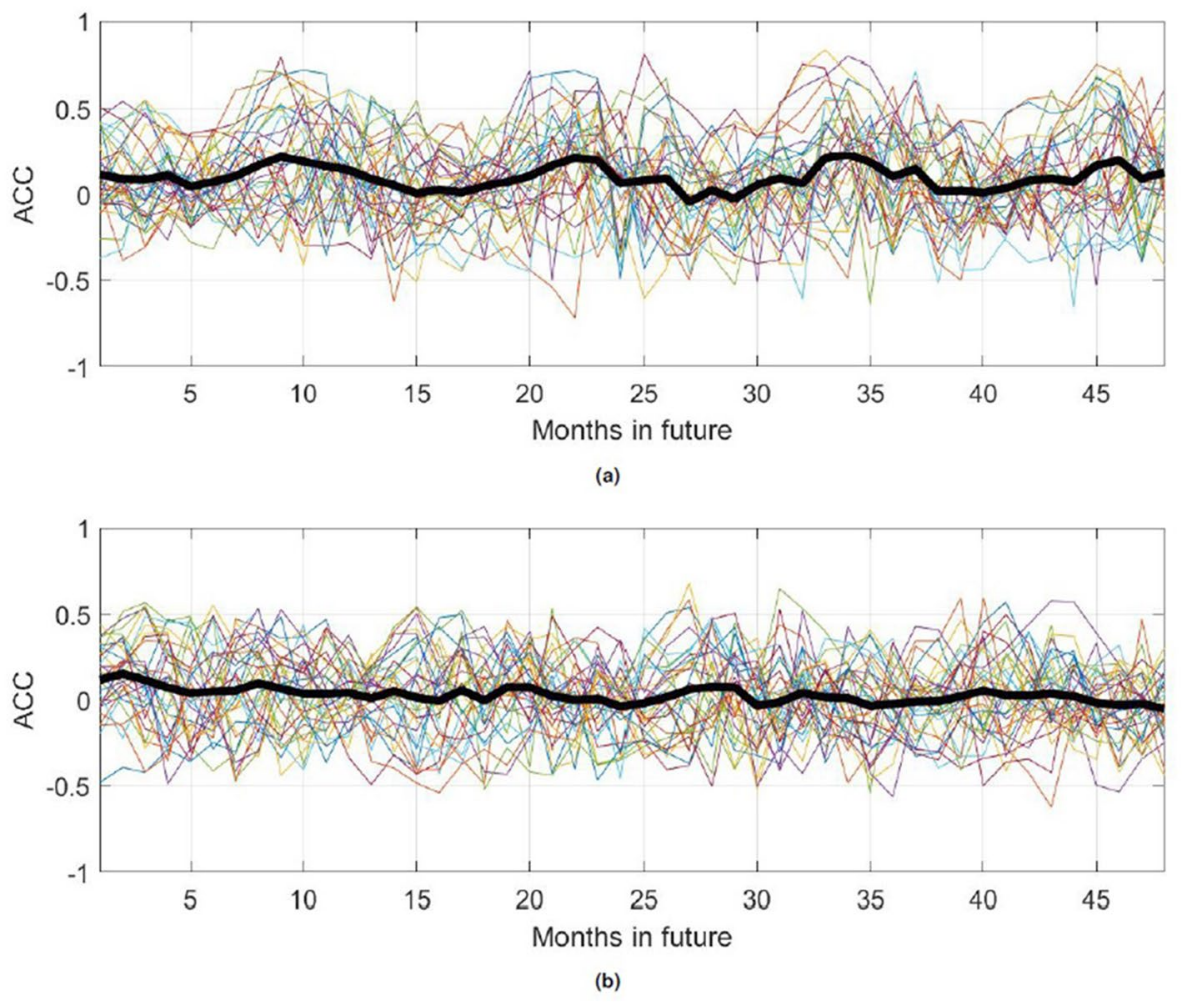
Anomaly correlation coefficient (ACC) values for four future years for the KMD prediction, for KMD applied to 37 4-year periods (1979–1982, 1980–1983, etc.). The thin, colored lines are monthly ACC values for each of the seven input periods and the thick black line is the mean of the monthly ACC values for all of the 37 input periods. (**a**) Northern hemisphere, (**b**) Southern hemisphere.

Turning now to the reference models based on the monthly means taken over the input period and on linear fits to the monthly values in the input period, Figs. 13, 14, 15, 16 show skill and RMSE values for KMD-based prediction compared to those reference models, as well as ACC values for the KMD-based prediction. The skill and ACC values are shown for each individual four-year input period as well as the mean over all of the individual traces, whereas for visualization clarity the RMSE results show representative errors for a single input period. Here results for each hemisphere are presented together because of their greater similarity as compared to the results above.

The mean skill for the KMD forecast versus the mean input of the data model is seen to be slightly positive for most future months, though a wide spread is evident in the traces for individual four year input periods, and the skill for the linear fit model case is seen to be consistently and increasing positive, as expected for a model whose design will produce an increasing error over time. These results are seen in the RMSE values, where the KMD prediction has comparable error to the input period mean model and increasing outperforms the linear fit model over time. The ACC values have slightly positive mean values, but as for the 30-year KMD input period results shown above they generally fall below the 0.5–0.6 synoptically useful threshold range.

## Discussion

These results show not only the previously-known existence of long term variation in sea ice concentration^27,28^, including long term decreases in sea ice coverage near West Antarctica and in the Arctic marginal seas, but also that a long-term exponential decrease in sea ice concentration exists and that Koopman Mode Decomposition allows a geographic view of where changes occur on annual and multi-year timescales. Such nonlinear trends in dynamics commonly result from positive feedback mechanisms such as those suggested in sea-ice dynamics studies^1,20,21,29^. The existence of long-term exponentially decaying modes seems to be of potentially substantial physical significance and could warrant further measurement (including other physical fields) and investigation.

A limitation of the application of KMD is that, as a purely data-driven tool, it does not provide the physical insight into the underlying forcing or other drivers of a system’s dynamical behavior that numerical or theoretical models can provide. In this case, the geographic heterogeneity of the sea ice concentration behavior in Antarctica suggests a possible link with a proposed physical driver of the decrease in the Antarctic ice mass balance. Recent work^30^ suggests the circumpolar deep water (CDW) flow as a physical mechanism for the decline of land-based ice due to increased glacier flow, where this increased decline is largest in the regions listed above where the decrease in the mean mode and increase in the annual variation mode is most apparent. The undersea topography of these regions is most consistent with the upwelling of relatively warm water by a strengthening CDW, leading to increase melting of ice shelves and, we suggest, reduced sea ice formation.

The accuracy of the predictions of future sea ice behavior by KMD reconstruction depends on the extent to which the sea ice concentration dynamics are governed by underlying nonlinear continuous processes, rather than stochastic or discontinuous drivers. That is, KMD-based prediction is expected to accurately predict variations in sea ice concentration due to the interactions of both long-term growth or decay and oscillatory behavior on fast or slow time-scales, however, changes due to “tipping points” such as the greater mixing between the Barents Sea and North Atlantic^31^ are not predictable from purely data-driven examination of sea ice concentration.

Taken in this light, the significantly different success of the KMD predictions for the Northern and Southern hemispheres suggest different sorts of underlying dynamics to the sea ice measurements. While the prediction results over multiple years for the Northern hemisphere do not display as high skill or ACC values as would be desired for future prediction purposes, the at least marginally positive values, particularly for the summer months, suggest that the observed recent sea ice dynamics are at least in large part a consequence of nonlinear long-term trends in the dynamical processes governing sea ice concentration behavior accessible to KMD when applied to the 30-year windows of data used for the KMD forecast and generally poorly represented by climatological models consisting of long-time averages of a non-stationary process. In contrast, the results for the Southern hemisphere indicate the dominance of oscillatory cyclo-stationary processes and thus are well represented, on average, by climatological models. Note that linear systems in the Koopman framework lead to a fixed set of frequencies that do not contain integer multiples (in the same way that Fourier decomposition of a periodic signal is linear if it does not contain harmonics of the base frequency). Since the annual phase averaged mode is equal to the Koopman mode at the annual frequency^7^, the success of climatological prediction in the southern hemisphere can be attributed to it behaving as a linear system with noise. The noise would in the Koopman framework be modeled by the continuous spectrum^32,33^ that we do not consider here.

## Methods

The objective of this study was to apply Koopman Mode Decomposition (KMD) and analysis techniques to existing satellite image data of sea ice concentration. Existing KMD algorithms were applied to the satellite images and the resulting Koopman modes and eigenvalues, defined in the following paragraph, showed the temporal and spatial details of the sea ice concentration (and anomaly) dynamics.

### Koopman modes and eigenvalues

The application of Koopman Mode Decomposition to time series data $g(t,\mathbf {z}_{0})\in \mathbb {R}^n\times I$, where *n* is the number of field observations, *I* the set of time snapshots and $\mathbf{z}_0$ an initial condition consists of expanding the time series observables onto the Koopman eigenfunctions to produce a set of Koopman modes and Koopman eigenvalues:^7,34^

$$\begin{aligned} g(t,\mathbf {z}_{0}) = \sum _{j=1}^{\infty } e^{\lambda _{j} t} \mathbf {v}_{j}(\mathbf {z}_{0}) + e^{\bar{\lambda }_{j} t} \bar{\mathbf {v}}_{j}(\mathbf {z}_{0}) +\mathbf{n}(t) \end{aligned}$$

where $\lambda _{j}$ are the Koopman eigenvalues, $\mathbf {v}_{j}$ are the Koopman modes, the overline indicates complex conjugation, and $\mathbf{n}(t)$ is part of the time evolution with continuous spectrum^7,34^. Note that the dependence on the initial state $\mathbf{z}_0$ is sometimes taken out of the mode itself, when eigenfunctions are used in the expansion. For each eigenvalue and its corresponding mode, the imaginary component of the eigenvalue $\mathfrak {I}(\lambda _{j}) = \omega _{j}$ determines the oscillation frequency $\omega _{j}$ of the mode *j* (and the oscillatory period $\tau ^{osc}_{j} = 1/\omega _{j}$) and the real component $\mathfrak {R}(\lambda _{j}) = 1/\tau ^{decay}_{j}$ determines the growth or decay time constant $\tau ^{decay}_{j}$ of the mode. It has been shown that short-timescale fluctuations can have a significant effect on large-scale features of climate systems, but the standard modeling practice of averaging the outputs of multiple simulation leads to the loss of this effect and thus incorrect predictions^35^. An advantage of KMD as a data-driven analytical technique is that in the eigenvalues it retains and uses the entire range of timescales present in the input data. The mode itself determines the spatial structure of the specific dynamical behavior given by the eigenvalue.

### Data pre-processing

The data pre-processing method was to convert the image data files from the NSIDC Sea Ice Index of sea ice concentration showing average monthly concentration to numerical arrays, remove the pixels corresponding to land areas, and reshape the remaining sea pixels into a 1-D array for each month. Note that there is a “polar data gap” in a circular region around the North Pole where concentration measurements are not available due to the coverage of the satellite based remote sensing instruments used to collect the sea ice concentration data. This region is traditionally either treated as completely ice covered or filled in based on the observed boundary conditions of the region^36^. For KMD, it is not necessary to fill in this region and so the points in the polar data gap were excluded from our analysis. The size of the polar data gap has decreased over time, however for consistency of analysis between different time periods we imposed the earliest, largest data gap on all years of data.

Data files were missing for a small number (three in the Northern hemisphere and two in the Southern hemisphere) of months in the 1980’s, so we chose to interpolate over the missing months to allow use of all data back to 1979, giving 40 full years of data (1979 to 2018).

The arrays for each month were then combined into a 2-D data matrix for performing KMD analysis run using KMD algorithms based on both Arnoldi^13^ and DMD type methods^16,17^. The results from the two categories of algorithms were found to be identical, which was taken to be a strong indication that the results are a good representation of the true Koopman eigenvalues and modes.

As described in the text, the calculated Koopman eigenvalues showed the time dependence (oscillatory and growth/decay) of the Koopman modes, which themselves showed the spatial structure of the time dependence of the input data.

To capture relatively short time scale dynamics, the analysis was performed on windowed data sets. The windowing consisted of performing KMD on subsets of the sea ice concentration data covering time periods of 5 to 40 years (e.g. five-year windows consisted of 1979–1983, 1980–1984, ..., 2014–2018).

The sea ice concentration data values $\mathbf {C}(x,t)$ (where we now explicitly show the dependence on location *x* and time index *t*) can be transformed to anomaly values $\mathbf {A}(x,t)$, where the anomaly is defined as $\mathbf {A}(x,t) = \mathbf {C}(x,t) - \mathbf {M}(x,i(t))$, where $\mathbf {M}(x,i)$ is the mean of the $i\text {th}$ calendar month at location *x* computed over the entire period of available concentration data (i.e., 1979–2018) and *i*(*t*) is the calendar month at time *t*. Note that the KMD analysis described herein can be applied to either $\mathbf {C}$ or $\mathbf {A}$.

### Forecast models

Five forecast models are used in this work: the KMD-based prediction and four reference models. Two of the reference models are the commonly used climatological and persistence models, whereas the other two models (the monthly mean over the input data and a linear fit to the monthly input data) were chosen to illustrate the influence of the relative cyclo-stationarity or nonlinear behavior of the system on the success of KMD-based prediction.

#### KMD-based prediction

Reconstruction of the $N_{p}$ sea ice concentration-related pixel values from $\mathbf {D}_\mathbf {k}$, where $\mathbf {D}$ represents either the sea ice concentration $\mathbf {C}$ or anomaly $\mathbf {A}$, at discrete time step *k* is performed using the Koopman eigenvalues $\lambda _{j}$ and the Koopman modes $\mathbf {v}_{j}$ obtained from applying KMD to the concentration values over *N* time steps (months, in this case):

$$\begin{aligned} \mathbf {D}_k = \sum _{j=1}^{N} \lambda _{j}^{k-1} \mathbf {v}_{j} \end{aligned}$$

Here, there are *N* Koopman eigenvalues and Koopman modes, where each Koopman eigenvalue is a single complex number and each Koopman mode has dimensions 1-by-$N_{p}$.

For $1 \le k \le N$, $\mathbf {D}_{\mathbf {k}}$ is termed a reconstruction of the *k*th time step in some arbitrary input data $\mathbf {D}$, as the Koopman eigenvalues and modes came from a decomposition of the observations over this time range and should simply reproduce the data used as input to the KMD. For $k>N$, $\mathbf {D}_{\mathbf {k}}$ is a prediction of the future behavior of the data for the (future) *k*th time step, based on the system dynamics deduced from decomposition of earlier observations. For the specific case examined here where $\mathbf {D}$ is either $\mathbf {C}$ or $\mathbf {A}$, we call the Koopman prediction $\mathbf {R}^{\mathbf {C}}$ or $\mathbf {R}^{\mathbf {A}}$, respectively.

The reported KMD forecast results were produced using *N* years of past data by applying KMD separately to the anomaly $\mathbf {A}$ for each calendar month, then performing prediction for the given calendar month. That is, for *N* years of past anomaly data and prediction of *M* years into the future, the 12*N* months of input data were separated into twelve sets, each set containing *N* months. KMD was applied once to each of the *N* data sets, producing twelve sets of *N* modes and *N* eigenvalues. A number $N_{\mathrm {recon}} \le N$ of modes and eigenvalues for each calendar month were used to predict the values of that calendar month for *M* future years using the KMD reconstruction equation given above, for time index $k = N+1$ to $N+M$.

The KMD forecast skill results for the 30-year input data period shown above were calculated by sorting the eigenvalue/mode pairs by the value of their residual, and calculating a KMD-based prediction as described above using the $N_{\mathrm {recon}}$ smallest residual pairs. The residual in effect measures the difference between a Koopman eigenvalue and mode pair calculated by an algorithmic implementation of KMD and the true eigenpair produced by the Koopman operator. Those eigenvalue and mode pairs with low residuals better represent the true dynamics of the system, and thus give better predictions of the system behavior at times beyond the time period of the input data. It was found that for forecasts using KMD applied to separate calendar months for 30 years of input data, $N_{\mathrm {recon}}$ values around 16 (out of $N=29$ total eigenpairs) produced the highest skill values. This can be understood as the exclusion of high-residual eigenvalue/mode pairs that can lead to inappropriately large growth in the forecast values beyond the time period of the input data.

No probability distribution is assumed in the KMD process so no statistical methods were applied. The deviation between the KMD reconstruction-based predictions of future sea ice concentrations and the actual values are due to two factors: the finite dimensionality of numerical realizations of KMD algorithms, which for relatively high-dimensional data as used in this study is not expected to be a major source of error, and the stochastic nature of the underlying climatological processes driving sea ice concentration dynamics, which will produce behavior not predictable in a purely dynamical model such as that produced by KMD reconstruction-based predictions.

#### Reference models

The two standard reference models used in this work were a climatological model and a persistence model. The climatological model $\mathbf {Q}$ predicts that the future value of $\mathbf {C}$ at a time *s* months in the future (relative to a time *t*) will equal the average value for that month of the year in the entire period of available data:

1 $$\begin{aligned} \mathbf {Q}(x,s+t) = \mathbf {M}(x,i(s+t)), \end{aligned}$$

where *i*(*t*) is the calendar month number (i.e., $1,2,3,\ldots ,12$) at a fixed time *t*. It is of interest to note that the climatological model is mathematically equivalent to a KMD model that uses the average mode and the phase-averaged annual mode^7^.

Secondly, the persistence model $\mathbf {P}$ predicts that the anomaly at the future month $s+t$ is given by

2 $$\begin{aligned} \mathbf {P}(x,s+t) = \mathbf {A}(x,t) \end{aligned}$$

where the time *t* is fixed and the index *s* of the future month is varied. This use of the persistence model is expected to produce low prediction errors for values of *s* that are small compared to the correlation time of the anomalies, and higher prediction errors for values of *s* that are large compared to the correlation time.

The two additional reference models used were an input data period mean model, $\mathbf {S}$, which predicts that the future values of concentration for each month will equal the average values for that month of the year in the input data period:

3 $$\begin{aligned} \mathbf {S}(x,s+t) = \mathbf {M}_{\mathrm {input}}(x,i(s+t)) \end{aligned}$$

where $\mathbf {M}_{\mathrm {input}}(x,i)$ is the mean of the concentration or anomaly, as appropriate, for the *i*th calendar month at location *x* computed over the time period of the KMD input data window, and a linear fit model, $\mathbf {T}$, which fits a separate first-order polynomial to the data for each calendar month in the input data period:

4 $$\begin{aligned} \mathbf {T}(x,s+t) = m(x,i(s+t)) k + b(x,i(s+t)) \end{aligned}$$

where *k* is the year number following the input data period and *m* and *b* are first-order polynomial coefficients, with dimensions $N_p$-by-12, calculated for each location *x* in the concentration or anomaly data over the time period of the input data.

### Prediction skill, mean square error and anomaly correlation coefficient

The definition used for prediction skill of the forecast model relative to a reference model is:

5 $$\begin{aligned} \text{ skill }(t) = 1 - \frac{\text{ MSE}_{forecast}(t)}{\text{ MSE}_{reference}(t)}, \end{aligned}$$

where MSE (the mean square error) for the forecast or reference is:

6 $$\begin{aligned} \text{ MSE }(t) = \frac{1}{N_p} \sum _{j=1}^{N_p} \left( E(j,t) \right) ^2, \end{aligned}$$

where *E*(*x*, *t*) is the error of the forecast or reference relative to the true value at location *x* and time *t*. The error for the persistence forecast is $E_\text {persist}(x,s+t) = \mathbf {P}(x,s+t) - \mathbf {A}(x,s+t)$ for *s* months in the future, while the error for the climatological forecast is $E_\text {clim}(x,s+t) = \mathbf {Q}(x,s+t) - \mathbf {C}(x,s+t)$. For the KMD forecast, the error is taken with respect to the appropriate truth value: $E_{\mathbf {R}^{\mathbf {C}}}(x,s+t) = \mathbf {R}^{\mathbf {C}}(x,s+t) - \mathbf {C}(x,s+t)$ and $E_{\mathbf {R}^{\mathbf {A}}}(x,s+t) = \mathbf {R}^{\mathbf {A}}(x,s+t) - \mathbf {A}(x,s+t)$, and similarly, for the input data period model $\mathbf {S}$ and linear fit model $\mathbf {T}$, the error is taken with respect to whichever observable the mean or linear fit was calculated over. A perfect forecast has a skill of 1, a model that performs exactly as well as the reference has a skill of zero, and a model that performs more poorly than the reference has a negative skill. Note that the root mean square error (RMSE) described in the text is the square root of the MSE.

The (centered) anomaly correlation coefficient (ACC) is:

7 $$ \text{ACC}(t) = \frac{\sum \limits _{j=1}^{N_p} [\mathbf {A}_{\text{model}}(j,t) - \bar{\mathbf {A}}_{\text{model}}(t)] [\mathbf {A}_{\text{truth}}(j,t) - \bar{\mathbf {A}}_{\text{truth}}(t)]}{\sqrt{\sum \limits _{j=1}^{N_p} [\mathbf {A}_{\text{model}}(j,t) - \bar{\mathbf {A}}_{\text{model}}(t)]^2 \sum \limits _{j=1}^{N_p} [\mathbf {A}_{\text{truth}}(j,t) - \bar{\mathbf {A}}_{\text{truth}}(t)]^2}}$$

where $\mathbf {A}_{\mathrm {model}}$ is the predicted anomaly from the forecast model and $\mathbf {A}_{\mathrm {truth}}$ is the true anomaly values. The sums are taken over the $N_p$ spatial elements in the region of regard (i.e., the number of pixels in the data images), and the overbar indicates a mean taken over the $N_p$ spatial elements. The ACC indicates the correlation between the forecast and true anomaly values, where a value of $+1$ at time *t* indicates perfect forecast quality at that time, zero indicates no correlation between the forecast and the truth, and $-1$ indicates an inverse (perfectly anti-correlated) relation between the forecast and truth.

## Supplementary information

Supplementary file1

## Data Availability

All data used in this work were obtained from the NSIDC Sea Ice Index^18^.
