# Supplementary material for: Exponentially decaying modes and long-term prediction of sea ice concentration using Koopman mode decomposition
Source: Sci Rep. 2020 Oct 1;10:16313. doi: 10.1038/s41598-020-73211-z (PMC7530978; doi:10.1038/s41598-020-73211-z)
Supplement: Supplementary file 1 — Supplementary file1 [file 41598_2020_73211_MOESM1_ESM.pdf]

Exponentially Decaying Modes and Long-Term Prediction of Sea Ice Concentration using Koopman Mode Decomposition

James Hogg, Maria Fonoberova, Igor Mezić

Appendix

An advantage of KMD for spectral analysis compared to the Fourier transform is the ability of KMD to detect spectral components with frequencies smaller than those detectable by the discrete Fourier transform (DFT). For regularly sampled data measured over a time period T, the frequency resolution of the Fourier transformed data is $\delta\omega = \frac{2\pi}{T}$. Frequencies less than $\delta\omega$ in the sampled data are not resolvable. In contrast, KMD does not have the limitation of fixed sized spectral domain bins, as Koopman eigenvalues have continuous values. Subject to the constraint that sampled measurements of oscillatory behavior are available from more than one spatial point and that a nonzero phase difference exists between those points, KMD can resolve frequency differences much smaller than the equivalent discrete Fourier transform frequency bin size.

We demonstrate this with a numerical experiment with two single frequency sinusoidal functions $f(t) = \sin(\omega t)$ and $g(t) = \sin(\omega t + \phi)$. Each function was sampled at a rate ω_s for $N = 100$ points, giving a total time period $T = N\delta t = \frac{2\pi N}{\omega_s}$ and a frequency resolution of $\delta\omega = \frac{\omega_s}{N}$.

Fig. 1 and Fig. 2 show comparisons of the DFT and KMD spectra resulting from different values of ω. The DFT spectrum is the magnitude of the result of applying the DFT to the time series produced by $f(t)$ (note that the same spectra would result from using $g(t)$, as the functions differ only by a phase shift, so the magnitude of their spectra are identical). The KMD spectrum is the result of applying KMD to input data formed from the time series produced by both $f(t)$ and $g(t)$, where this data has dimensions $2 \times N$, and so can be thought of as, for example, an image stack of N frames, where each frame is a two-pixel image. The KMD spectrum itself is defined as the imaginary components of the Koopman eigenvalues sorted in ascending order. Note that the DFT and KMD spectra in the figures show only the region of the spectra near zero frequency, in order to emphasize their differing behaviors in that region.

Fig. 1 shows the case for $\omega = \delta\omega$, where the DFT spectrum and KMD eigenvalue distribution both show the spectral component of the signal at $\pm\omega$, whereas for $\omega < \delta\omega$, shown in Fig. 2, only KMD properly resolves the spectral component at frequency ω.

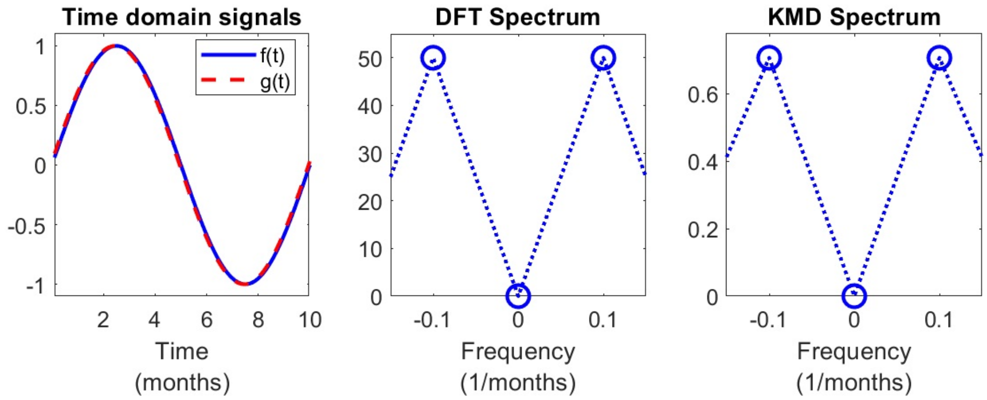

Figure 1. Example with oscillatory frequency ($\omega = 0.1$ 1/months) equal to the DFT frequency resolution. Left: The sampled functions. Middle: The DFT spectrum, showing the spectral components at plus and minus the oscillatory frequency. Right: The KMD spectrum (i.e., the imaginary components of the eigenvalues), also showing the spectral components at plus and minus the oscillatory frequency.

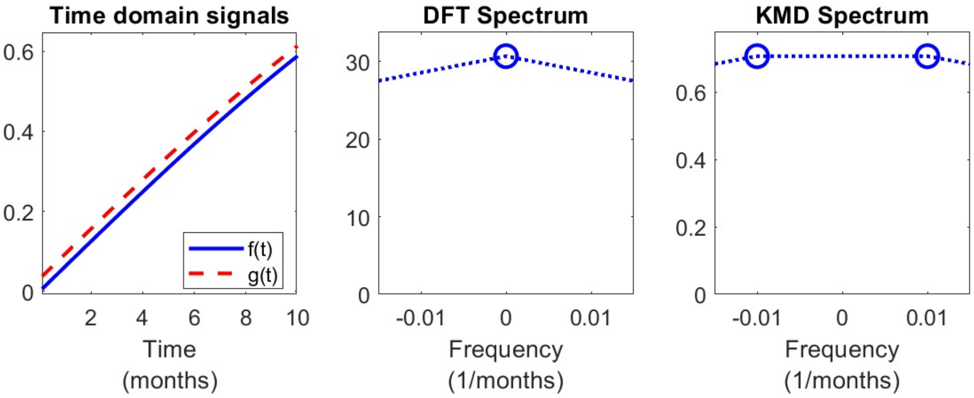

Figure 2. Example with oscillatory frequency ($\omega = 0.01$ 1/months) less than the DFT frequency resolution. Left: The sampled functions. Middle: The DFT spectrum, showing that the oscillatory frequency is not resolvable, as the spectral energy lies in the zero frequency bin. Right: The KMD spectrum showing that the spectral components at plus and minus the oscillatory frequency are still resolvable.
